# Exploring the barriers, facilitators, and opportunities to enhance uptake of sexual and reproductive health, HIV and GBV services among adolescent girls and young women in Zambia: a qualitative study

**DOI:** 10.1186/s12889-024-19663-8

**Published:** 2024-08-13

**Authors:** Alice Ngoma-Hazemba, Malizgani Paul Chavula, Noah Sichula, Adam Silumbwe, Oliver Mweemba, Mable Mweemba, Matildah Kakungu Simpungwe, Henry Phiri, Chowa Tembo Kasengele, Hikabasa Halwiindi, Margarate N. Munakampe, Joseph Mumba Zulu

**Affiliations:** 1 Department of Community and Family Medicine, School of Public Health, University of Zambia, Lusaka, Zambia; 2 Department of Epidemiology and Global Health, Umeå University, 901 87 Umea, Sweden; 3 Department of Community Education and Lifelong Learning, School of Education, University of Zambia, Box 32379, Lusaka, Zambia; 4 Department of Health Policy and Management, School of Public Health, University of Zambia, Lusaka, Zambia; 5 Department of Health Promotion, School of Public Health, University of Zambia, Lusaka, Zambia; 6 Ministry of Health, Ndeke House, Haile Selassie Avenue, PO Box, 30205 Lusaka, Zambia

**Keywords:** Reproductive health, Adolescents’ girls, Young women, Facilitators, Opportunities, Barriers

## Abstract

**Introduction:**

Adolescents and young women in low-middle-income countries face obstacles to accessing HIV, Sexual and Reproductive Health (SRH), and related Gender-Based Violence (GBV) services. This paper presents facilitators, opportunities, and barriers to enhance uptake of HIV, GBV, and SRH services among Adolescent Girls and Young Women (AGYW) in selected districts in Zambia.

**Methods:**

This study was conducted in Chongwe, Mazabuka, and Mongu Districts among adolescent girls and young women in Zambia. Key informants (n = 29) and in and out-of-school adolescents and young people (n = 25) were interviewed. Purposive sampling was used to select and recruit the study participants. Interviews were transcribed verbatim, and a content analysis approach was used for analysis.

**Results:**

The facilitators that were used to enhance the uptake of services included having access to health education information on comprehensive adolescent HIV and gender-based violence services. Non-governmental organisations (NGOs) were the main source of this information. The opportunities bordered on the availability of integrated approaches to service delivery and strengthened community and health center linkages with referrals for specialised services. However, the researchers noted some barriers at individual, community, and health system levels. Refusal or delay to seek the services, fear of side effects associated with contraceptives, and long distance to the health facility affected the uptake of services. Social stigma and cultural beliefs also influenced the understanding and use of the available services in the community. Health systems barriers were; inadequate infrastructure, low staffing levels, limited capacity of staff to provide all the services, age and gender of providers, and lack of commodities and specialised services.

**Conclusion:**

The researchers acknowledge facilitators and opportunities that enhance the uptake of HIV, GBV, and SRH services. However, failure to address barriers at the individual and health systems level always negatively impacts the uptake of known and effective interventions. They propose that programme managers exploit the identified opportunities to enhance uptake of these services for the young population.

**Supplementary Information:**

The online version contains supplementary material available at 10.1186/s12889-024-19663-8.

## Introduction

Two hundred million girls and young women in developing countries, that are desiring to avoid pregnancy, are not accessing and utilising modern contraceptive methods [1]. Adolescent girls aged 15–19 years in most countries report lower rates of satisfied demand for family planning compared to all women aged 15–49 years [1]. Over 50% of rural young women aged 15–24 years in sub-Saharan Africa experience pregnancy before turning 18 years [1]. In addition, the prevalence of HIV among adolescent girls is over a million; making it a leading cause of death for women aged 15–49 years globally [1]. One quarter of the Zambian population consists of adolescents who face several challenges related to their development, gender-based violence, unintended and teenage pregnancies as well as HIV and sexually transmitted infections, among others [2]. The impact of HIV, GBV and SRH challenges is disproportionately higher among adolescent girls due to many factors [2].

In Zambia, approximately 40% of girls and 45% of boys had first sexual intercourse between the ages of 15 and 19 years [3]. Adolescents are at a higher risk of indulging in unprotected sex resulting in unintended pregnancies and unsafe abortions [2], with the age group 15–24 years accounting for 40% of new HIV infections [2]. Gender norms and taboos around sexuality not only impede the capacity of adolescent girls and young women (AGYW) to protect themselves from HIV and GBV but also inhibit them from making informed decisions for themselves and their families [1]. Structural and systemic factors such as restrictive laws and policies discourage uptake of HIV, GBV and SHR services by adolescents [1]. In addition, stock-outs of preferred methods, lack of policy support for contraceptive provision in schools, negative attitudes among providers, criminalisation of sex before marriage, and social stigma further exacerbate adolescents’ inability to access these critical services [1].

Studies have shown that sexual and reproductive ill health, GBV, and HIV share similar root causes such as poverty, gender inequality, and social marginalisation [2]. Therefore, SRH, HIV and GBV should be addressed holistically, especially among adolescents and young women [4]. Evidence from SRH and HIV integration shows that it has benefits for both clients and service providers [1].

Interventions that aim to prevent HIV, and GBV and enhance uptake of SRH services include among others; social and behavioural change communication, Pre-Exposure Prophylaxis (PrEP), Voluntary Medical Male Circumcision (VMMC), cash transfers, multimedia and CSE to mention but a few [5]. These interventions target behavioral, biological, and structural factors that affect uptake of SRH, HIV and GBV services among adolescent girls and young women [6]. There have been several global commitments and huge investments by relevant stakeholders in providing these interventions in the past decades [5]. For instance, between 2017 and 2022 the Global Fund Strategy committed substantial resources to scaling up programmes to support AGYWs in thirteen countries [3] including Zambia and other Low And Middle-Income Countries (LMICs). However, AGYW in Zambia continue to face numerous challenges in accessing SRH, HIV and GBV services. In this study, the researchers explored the barriers, facilitators, and opportunities that enhance the uptake of SRH, HIV, and GBV services among adolescent girls and young women in Zambia.

## Methods

### Study setting and population

This study was part of a broader project titled "Formative Assessment of HIV, Gender-Based Violence, and Sexual and Reproductive Health Status among Adolescent Girls and Young Women in Zambia." The bigger study was conducted in 20 out of the 59 districts with high HIV prevalence. The target population comprised both in-school and out-of-school AGYW aged 10–24 years [7]. Data was also collected from government staff in the districts, NGOs, and community leaders involved in delivering HIV, GBV, and SRH services.

### Study design

This was a qualitative case study design that was conducted in Chongwe, Mongu, and Mazabuka Districts. These districts were sampled because they had high indicators relating to SRH, GBV and HIV affecting AYGW in Zambia. The districts were supported by partners and NGOs in collaboration with the Ministry of Health (MoH) in implementing interventions to mitigate challenges faced by programme managers and AYGW. The case study approach helped the researchers carry out an in-depth and comprehensive analysis of facilitators, opportunities, and barriers for increasing the uptake of SRH services among adolescent girls and young women [8]. This study adopted the approach and tools employed in a similar study titled “Sexual-risk behavior and HIV and syphilis prevalence among in- and out-of-school adolescent girls and young women in Uganda: A cross-sectional study” [3]. This research was conducted according to the consolidated criteria for reporting qualitative studies [4].

### Data collection and methods

The researchers conducted 54 interviews with AYGW and stakeholders (Table 1). They began by conducting 29 key informant interviews with district level stakeholders from Ministries of Education, Health and Social Welfare, NGOs, and community-based members such as parents of adolescents, traditional and church leaders.  These interviews sought to explore insights from key stakeholders who interact with and influence SRH service utilisation. Afterwards, they conducted 25 in-depth interviews with adolescents and young people who were in-and-out of school to understand their personal narratives and experiences in relation to SRH, HIV and GBV service uptake. They attained data saturation in all the interviews. Trained research assistants interviewed, transcribed, and coded the data in readiness for analysis.  The interviews lasted about 45-90 minutes. All participants were de-identified for anonymity and were assured that their access to SRH, HIV and GBV services would  not  be affected by their participation in the study.

**Table 1:** Participant information

Category	District 1	District 2	District 3	Total
**Stakeholders**				
District Officials	4	5	5	14
Implementing partners	2	2	2	6
Community leaders	2	2	2	6
Religious leaders	1	1	1	3
**SUB TOTAL**	**9**	**10**	**10**	**29**
**Adolescents And Young Women (AGYWs)**				
Female Sex Workers	2	3	2	7
GBV Survivors/ Child Abuse	3	3	3	9
Living with HIV (LWHIV)	3	3	3	9
**Sub total**	**8**	**9**	**8**	**25**
**TOTAL PARTICIPANTS**	**17**	**19**	**18**	**54**

### Data management and analysis

All interviews were transcribed verbatim and thereafter exported to NVivo 12 Pro Software for coding and analysis. The researchers employed content analysis techniques to analyse the data. The study team (ANH, MPC, MM) read through transcripts to understand facilitators, opportunities, and barriers to enhancing uptake of SRH services among adolescent girls and young women. Their focus was on HIV and GBV. The analysis team then generated a list of relevant codes from emerging themes to form a coding scheme following the strategies suggested by Maxwell [2]. Trained research assistants manually coded the transcripts. Description of the visible and obvious components of text and what it implied in the transcripts was considered in the coding process. Similarities and differences were closely examined to determine issues that commonly appeared in the transcripts [9]. The research team stored the data securely on computers that had passwords.

### Ethical considerations

Ethical approval to conduct the study was granted by the University of Zambia, Biomedical Research Ethics Committee (UNZABREC) (REF. 2460–202). Informed consent was obtained from all the participants, prior to participation, and the researhers ensured that the data obtained was confidential. For adolescents less than the age of 18 years, consent was sought from their parents for them to participate in the interviews while assent was also sought from the adolescents.

### Findings

The findings are organised around the barriers, facilitators, and opportunities for improving the uptake of SRH, HIV and GBV services. Each major thematic area, with its supportive subthemes, has been presented to provide a holistic picture of SRH, HIV and GBV services as they relate to adolescent girls and young women in Zambia as shown in Table 2 below.

**Table 2:** Major and Sub-themes

Major themes	Sub and explanatory themes
Barriers to uptake of HIV, GBV & SRH services	**Health systems barriers** • Lack of commodities • Lack of SRH services • Staffing challenges -trained human resource • Limited infrastructure • Long distance
	**Community/Individual barriers** • Myths & misconceptions about contraceptives • Gender and age of service providers • Delay in seeking SRH services • Social stigma • Concealing of GBV cases • Settling of GBV cases outside court • Cultural beliefs promoting GBV
Facilitators to uptake of HIV, GBV & SRH services	**Availability of services** • Family planning & counselling services • HIV/STI Testing & treatment • AYFS spaces providing SRH services • Community outreach & health promotion in schools • Integration of SRH services
Opportunities for improved uptake of HIV, GBV & SRH services	• Available organisations providing SRH services • Comprehensive sexuality education in schools • Stakeholder collaboration in SRH, HIV & GBV services

### Barriers to uptake of HIV, GBV & SRH services

Barriers to uptake of SRH, HIV and GBV services were described in the context of health systems, community, and individual levels. The following subthemes provide more details on the barriers.

### Health systems barriers

Health systems barriers included limited infrastructure, erratic supplies of essential commodities, lack of equipment, lack of trained human resources to provide specific SRH services, and limited access to services.

#### Infrastructure dedicated to a SRH services

There was limited or no space dedicated for adolescent services, which had a bearing on privacy during service provision, resulting in adolescents avoiding some health facilities. Further, limited infrastructure affected the scheduling of SRH services because some health facilities could not provide all the services in a day.

*“The infrastructure is a challenge…adolescents or peer educators don’t have permanent infrastructure where they can meet…usually it’s a shared space. Let’s say if they knock off from MCH…maybe that’s when the peer educators will go and access that building for them to start providing the services.” ****[KII: District Adolescent Focal Point, Female]***

### Trained human resource to provide SRH service

Some health facilities had limited personnel to provide SRH services to large communities in the catchment population. In some sites, the staff lacked training to provide services or had scheduling challenges.

*"These services…the providers need to be capacity built so that young boys and girls can have the confidence…so that when they go to the clinic, to access these services, the information cannot be spread to everyone (privacy), so it has to begin from there****.” [KII: NGO Staff]***

Some participants were concerned with the lack of consistency in service provision:

*“You will find nurses who say, come tomorrow, or direct you see the doctor. And in such health facilities, I wish they could employ people such that even in the night they are working at the GBV side, it can make a lot of sense.” ****[IDI: Sex worker, Female 23 years]***

### Supplies and essential commodities for SRH services

Frequent shortage of essential commodities like condoms and contraceptives negatively affected the choice of methods to prevent unwanted pregnancies and sexually transmitted infections:

*“…the staff will tell you that they do not have the condoms at the facility at that particular time, so you will be told to come at a later time…but some people buy from the shops.” ****[IDI: PLWHIV, 20-24 years]***

### Individual and community barriers

Side effects of drugs and hormonal contraceptives, and delay in seeking of care were reported by some participants. At the community level, it was common for participants to indicate that gender and age of health care providers made it difficult for young people to seek SRH services. Social stigma, concealing and hiding of GBV cases, and settling of GBV cases outside court were some of the cultural practices that promoted GBV and discouraged uptake of SRH services.

#### Side effects of contraceptives

Side effects such as prolonged menstrual bleeding and weight gain were some of the reasons adolescents shunned hormonal contraceptives.

“*Family planning has so many side effects, as a result some sex workers may stop accessing it and switch to use of condoms because some make them bleed throughout, how can one sleep with a person if they are in such a condition? …this family planning reacts differently, for some it’s working well while for other people it gives them challenges…” [IDI: Sex Worker and GBV, 20 Years].*

#### Gender and age of health service providers

Female adolescents were not free to access SRH services from a male provider:

*“The challenges I have experienced is that most adolescents will be closed especially if they find a male health care provider…they take time to open up their challenges…now to find time to go and make another appointment…” [KII: Facility Adolescent Focal Point, Male].*

Further, adolescents who are victims of GBV may not fully be able to disclose their experiences if they think the provider is not of appropriate age:

*“…sometimes someone can have a problem with GBV, but they don’t find a provider in their age-group to express themselves…when they find old people there, it is a barrier. So even the Youth friendly corners we include young people in those clubs because there is a challenge when an older man or woman is found at the facility, they cannot express themselves.” [KII* *:* *PTA Chairperson].*

Adolescents who were sexually abused delayed to seek care because they were afraid of being victimised by some family members:

*“So, you find that by the time this victim will be reaching the hospital and be examined…the evidence is compromised. Sometimes you would find that the doctor is going to say, but I cannot see anything here … Meanwhile the perpetrator will even know that he defiled that young person, and you know when it comes back again to us and the courts, we go for what is documented here. You would find that the perpetrator would even be acquitted* ***.” [KII: Facility Adolescent Focal Point, Male].***

#### Social stigma

Social stigma was experienced both at individual and community level. Some adolescents were afraid that providers would associate being HIV-positive with promiscuity and some parents did not disclose the HIV positive status of their child for fear of stigmatisation: *“When people hear, to say someone is HIV positive, the first thing that comes into their minds is ‘sienzomvela’ [being promiscuous) you know. A person was a prostitute, sleeps around with different guys, but it is a different case for me.” [IDI: Living with HIV, 18 Years].*

#### Concealing of GBV cases

Some families protected GBV perpetrators from being arrested, especially if they were breadwinners and tended to report these cases to the police very late: *"…They are a lot…we had a case of an 18-year-old who was sexually abused, but they never reported and did nothing about it…and those are some challenges that we face. She is not the only one, there are many girls who get pregnant, but the parents don't report as a result many people are doing the same thing without fear and they are not being reported due to fear from the community." [Health Worker-One Stop Centre].*

A social worker added: *“Yeah, I think it’s a challenge because we still have many cases where a child is defiled and never talked to anyone. And that person defiling will keep on doing it…not until parents or the guardians just come on their own.” [Social Welfare].*

#### Settling of GBV cases outside court

It was common to hear that some cases of defilement were settled by church leaders as opposed to reporting to the police: *“Even through these church leaders, they would rather not go to the police, but they go for counselling at the church, yes…or to the groups that provide similar services” [In-School, Males 15 – 19 years].*

Some parents did not report defilement cases for fear that the community would question their parenting style: *“They don’t want to be judged by the community …for example if a girl is raped the community will question the upbringing of that child, and that’s the reason why people keep it within the family.” [IDI: PLWHIV, 20-24 years].*

#### Cultural beliefs that impede uptake of GBV and SRH services

Some traditional and cultural beliefs promote and sustain GBV against adolescent girls and young women. Married adolescents are encouraged to accept some form of GBV by spouses and partners to show loyalty and obedience:

*“…lady could be beaten by her spouse/partner, she won’t share because it is taboo in our culture. It is considered love when your husband is beating you… we need to change the mindset of our people, and what I have emphasised is that there are certain traditions which are good and some are bad. We need to embrace good ones, those which are bad we discard them, and how are we going to judge those traditions which are bad?”[KII: Chief]*

Some parents beat their adolescents or throw away the contraceptives if they find them in their possession. For this reason, they chose not to go against their parents for fear of being chased from home. In addition, adolescents are afraid to go to health facilities because they are frowned upon for accessing contraceptive services.

*“Some parents beat when they realise that their child has started accessing family planning services. Once they find those contraceptive pills, they will throw them and beat the girl as a result that girl will stop accessing the service because they are scared that if their parent come to know it will turn out to be a big issue. Due to this reason, the adolescent will opt not to go against their parents because their parents are still keeping most of them. Fearing that the community will laugh and point fingers at them that they are too young for them to start accessing such services.” [IDI: Adolescent married/pregnant at 19 years, PLWHIV]*

### Facilitators to uptake of SRH services

The uptake of SRH services facilitators were described in relation to accessibility and integration of services provided at the health facilities, respectively. Further, this section highlights the importance attached to community outreach services, health promotion in schools, integration of SRH services into other services and programs, and GBV referral systems, community linkages, and community-people-centered health service approach.

### Accessibility to SRH services

Accessible services for comprehensive adolescent SRH were family planning counselling and contraception; HIV/STI counselling, testing and treatment including ART; adolescent and youth-friendly spaces (AYFS) and SRH information.

#### Family planning counselling and contraception

For adolescents, family planning and contraceptives services were provided from public health facilities, and some private health institutions, including commercial pharmacies. Most adolescents were aware of the services and even accessed them. An adolescent and commercial sex worker explained that; “*When we go to the clinic for family planning, they teach us that we should be using condoms…and this other thing which came recently [PrEP*].” *[Sex Worker; 20 years].*

The benefits of accessing these services at public health facilities included counselling and teaching provided by the health care providers. An adolescent who experienced early pregnancy and was living with HIV explained; *“Adolescents should go to health facilities to access family planning services, because they will advise them on what type of contraceptives to take and how to take them.****”**** [Adolescent married/pregnant at 19 years, PLWHIV].*

#### HIV/STI Counselling, testing and treatment

The HIV and STI counselling, testing and treatment services for adolescents, regardless of age and sex were provided at the health facilities. ART services were always available to adolescents when they visited the health facilities, and this was acknowledged by adolescents and sex workers. Those who tested HIV positive were enrolled into support groups that talked about nutrition and treatment for people living with HIV. The following narratives were common:

*“It is good to be tested because I know how my life is doing. Since we are always found in the bars, sometimes we get drunk, often sometimes a man is not wearing a condom, and then he uses you with vigor. Therefore, that is why after three or two months I am tested. I am found with people who encourage me to get tested.” ****[Sex Workers, 15-19 years]***

#### Adolescent and youth-friendly spaces

The participants explained that the Adolescent and Youth-Friendly Spaces (AYFS) played an important role in promoting access to SRH information and counselling services. The services that were provided were adolescent-friendly HIV counselling services and treatment of sexually transmitted infections. Everyone, including learners, were accommodated for family planning services and information because SRH services were offered by fellow peers, who were accommodative and non-judgmental. The following were familiar expressions from the adolescents in and out of school:

*“So, in terms of sexual and reproductive health services, like I said, we have youth friendly spaces where youths can access contraceptives like condoms even injectables, they are also able to access health information talks, they can also receive referrals maybe to specialized treatment. …and they can also receive treatment if they have STIs and any ailments, where they are not freely able to disclose elsewhere” ****[In School-Girls, 15 – 19 years]***

Further, adolescents found it easy to speak with their fellow young people because they do not feel judged: *“I will go for an adolescent because they are my age mates, therefore it is very easy for me to interact with them and because we are of the same age, we understand each other. They will not judge me…they are old (health care providers), it is difficult to interact with them on such matters. We are trained/socialized not to have such* conversations (sexuality issues) *with elderly people.”* **[GBV Survivor, 15-19 years, female**].

#### Community outreach services and health promotion campaigns in schools

Through community-based outreach services provided by health facility staff and Non-Governmental Organisations (NGOs), adolescents were encouraged to access HIV/STIs, SRH and GBV services. For example, adolescents were tested at the school premises and those that were found with HIV or any other STIs, were referred to the health facilities for further attention. In addition, community outreach programmes use adolescent school structures such as clubs and associations to reach this population group. Through these structures, adolescents are taught various SRH topics such as the dangers of unprotected sex resulting in HIV and other STIs transmission. Information on access to HIV treatment and GBV services was also provided. The following participants added to these narratives:

*“Of course, we have got different programs, for example football, because we are also into games ...yes. There are women, young adolescents, adult men, and when we are there, they teach us about HIV and other programs… At least everyone should know their status, when you are found positive, they will put you on medication. If you’re found negative, they will know the measures to take.” [Stake Holder; Red Cross, Female]*

*“At school, they taught us that HIV is a disease that is transmitted for example, by using a razor blade used by the person who is HIV positive…if there is blood contact you can also be infected. Apart from that, if you are a female and you have sex with a man who is HIV positive without protection, you can be infected with the HIV virus.” [IDI; Sex Worker and GBV survivor, 20 Years]*

#### Integration of SRH services into other health services and programs

Integration of SRH education into sports activities and youth clubs facilitated the dissemination of SRH services and information to adolescents. Further, SRH integration into health services such as HIV and GBV allowed for capturing adolescents that may otherwise be reporting for other services. Integration of SRH into the adolescent school programmes included activities such as teaching adolescents about SRH and building the capacity of healthcare providers, teachers, and the police. These capacity-building programmes focused on activities such as training the aforementioned to provide counselling, screening, and treatment services to adolescents regarding various health issues, including cancer screening, HIV, GBV and other SRH services. A key informant working at one-stop center had the following to say:

“*…these programmes are already integrated, I will give an example, we don’t work during the weekend, and so may be at night, in the ward like this morning, a child came who is 14 years and already pregnant. They came for antenatal services, and the officers noticed that this child is young, so they referred them to one stop center, and we captured that case, and it was a case of defilement by a brother or cousin within the same home.”* *[Partner-Legal; One stop Centre, Male]*

#### Community and people-centered health service approach

One major facilitator in the provision of HIV, GBV, and SRH services to adolescents was the community and people-centered approach to service delivery. Organizations that embraced these approaches to provide HIV, GBV and SRH services to adolescents were; CIDRZ, Child Fund and Santu. Services provided included HIV information and education, community nutrition programmes and engaging community leaders and parents. A key informant at World Vision shared his thoughts on collaborations.

*“…we meet in the community… doing HIV related activities in the district…I am aware of… CIDRZ. Then we also have Child Fund, they are also doing reproductive health, yeah, as we are doing here. There is also Santu which has come on board, but I don’t think they are doing HIV, but more into nutrition….” ****[Partner, World Vision]***

### Opportunities to uptake of HIV, GBV SRH services

Some of the opportunities identified to improve the uptake of SRH services were availability of organisations providing the SRH services, the introduction of comprehensive sexuality education, collaboration among stakeholders in serving adolescents and young women, and strengthened community referral systems.

#### Youth organisations providing SRH services

Some youth NGOs supplemented government efforts in providing SRH services and information to adolescents because of a huge demand for adolescent SRH services. They had structures within the communities where adolescents accessed SRH services such as family planning. However, adolescents were also free to visit these offices to ask questions concerning SRH. In addition, adolescents were referred to appropriate institutions in situations where the NGOs were not able to provide services. A community leader added:

*“… there are organizations like Youth Achievers, one can go to this office and ask questions … can access some services like condoms…then also you can go to health posts like Kabengele (local name) clinic or Kakoswe Hospital*.” *[Chief]*

#### Comprehensive sexuality education in schools

Comprehensive Sexuality Education (CSE) is available for adolescent learners in school from grade 5. Age appropriate SRH topics progress as the student grows, but also other topics such as social studies and the inviting of police to talk to adolescents helps to complement the provision of SRH information in schools. The guidance and counselling office provides a platform that counsels the students in conjunction with the teachers. A Development Officer had the following to say:

*“We also trained teachers who are not guidance teachers, but they're teaching subjects that have the component of sexuality such as science, those that teach social studies, and those that teach Home Economics….we train them so that as they are teaching those subjects, they even take the component of sexuality education, but we also just do it as guidance teachers…we also have health education topics as standalone topics, so we do teach them sexuality education.”* *[Government staff]*

#### Collaboration among stakeholders in the provision of SRH and GBV services

Collaboration among various cross-cutting sectors provided an opportunity for linking or referring of services among different stakeholders by leveraging on their individual strengths and capacity in the provision of SRH, HIV and GBV services. For example, there were NGOs that offered shelter to GBV victims and survivors as well as special services for children with disabilities. Some organisations also created awareness of GBV and drug abuse, by sponsoring programmes on radio, in communities, churches, and schools. Further, there was coordination among various departments offering GBV services and they also escalated the referral to the next level of care. Coordination among the stakeholders in the provision of services such as SRH services avoided duplication.

*“What has worked well in this is coordination, like I said, stakeholders have partitioned the district. Rather than concentrating on one place and duplicating the work, one stakeholder deals with a certain area so that at least we cover as much ground as possible. The other benefit is to know who is doing what so that you don’t waste resources. So, if stakeholder is dealing with information, you work with those instead of starting all over again.”* *[Legal One-Stop-Centre, Male]*

In addition, a sex worker added: *“There are these people who use motor bikes in their work, they work on adolescent issues to do with GBV…USAID…they are the ones who got me and took me to a hospital known as a one-stop center, it was called as ASAZA, now it is called one-stop center. Then that’s how they helped us with care.” [Sex Worker Female, 23 years].*

In the same setting, the social welfare officer added: *“So what do you do to the child…move the child to a safe place and find alternative care or we are guided on how to prepare those children as child witnesses if the case is going to court. It also helps us to prepare the committal orders and what not, until the child is placed under alternative care …Yes, some cases have been to courts of law and have been closed. You know these cases you can’t start citing examples…but we have some cases yes.” [Social welfare officer, Female].*

#### Strengthened SRH and GBV referral systems and linkages

The presence of strong referral systems and linkages facilitated the provision of SRH services. For instance, participants pointed out that there were mechanisms available to identify and refer adolescents who experienced GBV in the communities. Further, the follow-up mechanisms for the referred cases helped to guarantee the health of the adolescents or the victims. In addition, the study districts’ existing linkages to legal services, including safe spaces and homes for GBV survivors, facilitated the provision of these SRH services. A guidance teacher explained how they followed up on the cases they referred with success below:

“*Most of the cases which we have referred, they appear to be successful because we make a follow up, we follow the children, we find out, how did you move, because we work in conjunction with the health facilities that we have…. is a child healthy…. they call it a corner, girl child friendly corner. When we take them there, they usually counsel them and sometimes they will even give us feedback*.” *[Guidance Teacher, Female]*

## Discussion

This study explored barriers, facilitators, and opportunities to the uptake of SRH services, focusing on HIV and GBV services. While it was common to hear participants explain what would facilitate uptake of SRH services in the context of limitations in the study settings, this study highlights the need for a client sensitive approach to comprehensive service provision for adolescents and young women. Against this backdrop, the government has over the past few decades developed and revised the Adolescent Health Strategic Plan to address the barriers to uptake of SRH services while providing opportunities for improved service delivery for this population group [4, 20]. The researchers note that strong referral networks, service provision by age peers or near peers, and the co-delivery of multiple SRH and GBV services will have a multiplier effect on the uptake of services and the improved health of AGYWs in Zambia.

The study revealed that the availability of SRH, HIV, and GBV services including family planning, counselling, HIV/AIDS counselling, testing and treatment, and youth-friendly services facilitate the delivery and uptake of the services by adolescents. For instance, HIV prevention is key to reducing the incidence among adolescents and young people. The findings suggest the availability of integrated HIV and STI counselling, testing, and treatment services in some health facilities that are accessible to nearly all adolescents regardless of their age and sex. This co-delivery of multiple SRH and HIV services resonates with the evidence that suggests that this approach could have led to a slight increase in the uptake of HIV Testing and Counselling among youths, especially young women in Zambia [10].

On the other hand, SRH service delivery and uptake among adolescents and youth is still low in Zambia. Studies conducted in Western Africa found that health system structures play a significant role in supporting a continuum of care through provision of friendly SRH services, demand creation and enhanced social accountability to deliver them effectively [1]. However, the researchers also observed that different SRH, HIV and GBV services including education and counselling for contraceptives, HIV/AIDS counselling, testing, and treatment were either inadequate or inaccessible to some adolescents and young people in the communities that were studied. In Uganda, for example, poor social and economic status appears to be one of the determinants contributing to reduced uptake of the services [2].

Therefore, the lack of accessibility and utilisation of SRH contributes to unplanned pregnancies and can have far-reaching consequences on the health, social, and economic well-being of adolescents [11]. In sub-Saharan Africa, many of the adolescent girls who fall pregnant never go back to school, and pregnancy is highlighted as the major cause of school dropout further widening the social inequalities [2]. For example, service obstacles that adolescents face in Zambia have been reported and they include limited contraceptive options, compounded by judgmental attitudes of providers, lack of confidentiality, and a lack of policies and guidelines for protecting adolescents’ rights to information and services [4]. Therefore, such obstacles can be mitigated by reinforcing the peer-to-peer service delivery, although more studies have recommended further understanding of the complex SRH interventions and their interactions with local health systems [9]**.**

Although present in some of the districts, referral networks in the delivery of GBV services such as integrated and strengthened linkages to health systems remain weak in Zambia. For example, the collaboration among different players including government departments, healthcare workers, peer educators, community, and traditional leaders in the delivery of SRH, HIV and GBV services is weak. For example, the researchers observed that in a number of settings, GBV cases were concealed at community level and settled outside the formal justices. This was because of the social and economic factors associated with stigmatisation and economic loss due to possible arrest of the breadwinner of the victim and survivor of GBV. However, the researchers noted that parallel implementation of SRH and GBV programmes and strengthened referral networks provided survivors of gender-based violence (GBV) real-time access to safe, and confidential services that support their immediate and long-term health, healing, and empowerment [12]. Evidence suggests that adolescent peer-to-peer strategy in the delivery of contraceptives, HIV counselling, testing, and treatment was associated with acceptability and uptake of services and contributed to improved health decision-making [2]. Furthermore, community empowerment programmes targeting adolescents and young people are essential in enhancing their capabilities including finances to support access to SRHR, HIV, and GBV services from a health facility of their choice. However, some of them fail due to social, cultural, and religious barriers. Therefore, the community participatory interventions that engage parents, community pharmacies dispensing contraceptives, and traditional and community leaders to support the delivery of adolescent and youth-friendly services are crucial.

Further, integration of SRH, HIV, and GBV services in the media, schools, community pharmacies, markets, villages, sports activities, and bars can help to enhance community acceptability of these services [13–17]. On the other hand, the involvement of key actors including adolescents promotes community ownership and sustained delivery of services [16, 18, 19]. It is documented that the engagement of stakeholders, which includes service users helps to change their attitudes towards such services and is key to improved uptake of services [20]. As observed in other studies, the researchers documented deep-rooted social-cultural practices that discourage communication about sexual issues with adolescents [21]. Additionally, comprehensive delivery of SRH services such as contraceptive options is hindered by policies and guidelines that restrict adolescents from accessing SRHR information and services [12]. The researchers noted that effective use of the school systems may play a significant role in addressing the restrictive accessibility to SRH information and subsequently promoting better linkages of adolescents to adolescent youth-friendly services [22, 23]. The school and health system collaboration promotes and offers hope for the effective implementation and improved SRH service delivery and uptake especially among adolescents [12]. However, there is a need to increase teachers’ capabilities in the delivery of SRH education and reduce their discomfort [12].

### Limitations and strengths of the study

Although the researchers did not conduct a policy analysis of the health systems delivery landscape to understand how the policy environment affects barriers, facilitators, and opportunities to delivering and accessing SRH, GBV, and HIV, sampling from a wider range of districts enabled us to comprehensively view the context of challenges faced during implementation of the services.

## Conclusion

The study identified barriers to accessing SRH, HIV and GBV services among adolescent girls and young women in Zambia at individual, community, and health system levels. The barriers included cultural and social factors, limited infrastructure, and weak health system responsiveness. To address these barriers, we recommend implementing interventions that can promote strong collaboration among stakeholders, strengthen linkages to SRH services, and promote collective action in delivering SRH services at community level. Applying a collaborative governance approach to policy adoption, implementation, and service delivery is crucial to improving service utilization among adolescents and youths given the multi-sectoral nature of SRH, GBV, and HIV services. Future studies should analyse the policy context in which SRH, HIV, and GBV services are delivered as the context plays a significant role in influencing the delivery and uptake of the service.

### Supplementary Information

Supplementary Material 1

Supplementary Material 2

Supplementary Material 3

## Data Availability

The data shall be made available upon request.

## Abbreviations

UNZABREC: University of Zambia Biomedical Research Ethics Committee
NHRA: Zambia National Health Research Authority
IPs: Implementing Partners
MoE: Ministry of Education
MoH: Ministry of Health
COVID-19: Coronavirus Disease 2019
SRH: Sexual and Reproductive Health
GBV: Gender-Based Violence
HIV: Human Immunodeficiency Virus
SRHR: Sexual and Reproductive Health and Rights
USAID: United States Agency for International Development
CIDRZ: Centre for Infectious Disease Research in Zambia
ART: Antiretroviral Therapy
AYFS: Adolescent and Youth-Friendly Spaces
PLWHIV: People Living with HIV
NGOs: Non-Governmental Organizations
PrEP: Pre-Exposure Prophylaxis
VMMC: Voluntary Medical Male Circumcision
AGYW: Adolescent Girls and Young Women
ZDHS: Zambia Demographic and Health Survey

## References

[1] Ahun MN, Aboud F, Wamboldt C, Yousafzai AK. Implementation of UNICEF and WHO's care for child development package: Lessons from a global review and key informant interviews. Front Public Health. 2023;11:1140843. 10.3389/fpubh.2023.1140843 PMC9978394 36875409

[2] Akazili J, Kanmiki EW, Anaseba D, Govender V, Danhoundo G, Koduah A. Challenges and facilitators to the provision of sexual, reproductive health and rights services in Ghana. Sexual and Reproductive Health Matters. 2020;28(2):1846247. 33213298 10.1080/26410397.2020.1846247 PMC7888097

[3] Black MM, Hurley KM. Early child development programmes: further evidence for action. Lancet Glob Health. 2016;4(8):e505–6. 27443769 10.1016/S2214-109X(16)30149-8

[4] Barnhart DA, Farrar J, Murray SM, Brennan RT, Antonaccio CM, Sezibera V, et al. Lay-worker delivered home visiting promotes early childhood development and reduces violence in Rwanda: a randomized pilot. J Child Fam Stud. 2020;29:1804–17. 10.1007/s10826-020-01709-1

[5] Cuartas J, McCoy D, Sánchez J, Behrman J, Cappa C, Donati G, et al. Family play, reading, and other stimulation and early childhood development in five low‐and‐middle‐income countries. Dev Sci. 2023;26(6):e13404. 10.1111/desc.13404 PMC11475363 37114644

[6] Bach B, Stefaner M, Boy J, Drucker S, Bartram L, Wood J, et al. Narrative design patterns for data-driven storytelling. Data-driven storytelling: AK Peters/CRC Press; 2018. p. 107–33.

[7] Musonda P, Halwiindi H, Kaonga P, Ngoma-Hazemba A, Simpungwe M, Mweemba M, et al. HIV, syphilis and sexual-risk behaviours’ prevalence among in-and out-of-school adolescent girls and young women in Zambia: a cross-sectional survey study. PLoS ONE. 2024;19(6):e0294545. 38837995 10.1371/journal.pone.0294545 PMC11152284

[8] Daelmans B, Black MM, Lombardi J, Lucas J, Richter L, Silver K, et al. Effective interventions and strategies for improving early child development. BMJ : British Med J. 2015;351:h4029. 26371213 10.1136/bmj.h4029

[9] Cavallera V, Tomlinson M, Radner J, Coetzee B, Daelmans B, Hughes R, et al. Paper 5: Scaling early child development: what are the barriers and enablers? Arch Dis Child. 2019;104(Suppl 1):S43. 30885965 10.1136/archdischild-2018-315425 PMC6557300

[10] Zulu JM, Mwamba T, Rosen A, Matenga TFL, Mulanda J, Kaimba M, et al. Community engagement for the Voluntary Medical Male Circumcision (VMMC) program: an analysis of key stakeholder roles to promote a sustainable program in Zambia. Gates Open Research. 2022;6(50):50. 37069966 10.12688/gatesopenres.13587.1 PMC10105033

[11] Greene ME, Stiefvater E. Social and gender norms and child marriage. Advanced Learning and Information on Gender Norms. 2019. [cited 13th June 2024]. Available from: https://www.alignplatform.org/sites/default/files/2019-04/align_child_marriage_thinkpiece.pdf.

[12] Jeong J, Franchett EE, Ramos de Oliveira CV, Rehmani K, Yousafzai AK. Parenting interventions to promote early child development in the first three years of life: a global systematic review and meta-analysis. PLoS Med. 2021;18(5):e1003602. 10.1371/journal.pmed.1003602 PMC8109838 33970913

[13] Chavula MP, Matenga TFL, Halwiindi H, Hamooya C, Sichula N, Jones DL, et al. Factors shaping responsiveness towards sexual gender-based violence during the COVID-19 Pandemic in Africa: A systematic review. Cogent Public Health. 2023;10(1):2234600. 10.1080/27707571.2023.2234600

[14] Zulu JM, Sitali D, Shroff ZC, Lamba G, Sichone G, Michelo C, et al. Barriers and facilitators for integration of guidelines on operating health shops: a case of family planning services. J Pharmaceut Policy Pract. 2021;14(1):1–11. 34784959 10.1186/s40545-021-00337-4 PMC8594102

[15] Tetui M, Hurtig A-K, Jonsson F, Whyle E, Zulu J, Schneider H, et al. Strengthening research and practice in community health systems: a research agenda and manifesto. Int J Health Policy Manag. 2022;11(1):17. 34380193 10.34172/ijhpm.2021.71 PMC9278394

[16] Zulu JM, Chavula MP, Silumbwe A, Munakampe MN, Mulubwa C, Zulu W, et al. Exploring politics and contestation in the policy process: the case of Zambia’s contested community health strategy. Int J Health Policy Manag. 2022;11(1):24. 34814675 10.34172/ijhpm.2021.145 PMC9278392

[17] Schneider H, Olivier J, Orgill M, Brady L, Whyle E, Zulu J, et al. The multiple lenses on the community health system: implications for policy, practice and research. Int J Health Policy Manag. 2022;11(1):9. 34273937 10.34172/ijhpm.2021.73 PMC9278387

[18] Matenga TFL, Zulu JM, Moonzwe Davis L, Chavula MP. Motivating factors for and barriers to the COVID-19 vaccine uptake: a review of social media data in Zambia. Cogent Public Health. 2022;9(1):2059201. 10.1080/27707571.2022.2059201

[19] Sialubanje C, Sumbwa PI, Zulu N, Mwanza NB, Chavula MP, Zulu J. Gender integration and female participation in scientific and health research in Zambia: a descriptive cross-sectional study protocol. BMJ Open. 2023;13(3):e064139. 36878653 10.1136/bmjopen-2022-064139 PMC9990657

[20] Mutea L, Ontiri S, Kadiri F, Michielesen K, Gichangi P. Access to information and use of adolescent sexual reproductive health services: Qualitative exploration of barriers and facilitators in Kisumu and Kakamega, Kenya. PLoS ONE. 2020;15(11):e0241985. 33180849 10.1371/journal.pone.0241985 PMC7660470

[21] Belay HG, Arage G, Degu A, Getnet B, Necho W, Dagnew E, et al. Youth-friendly sexual and reproductive health services utilization and its determinants in Ethiopia: a systematic review and meta-analysis. Heliyon. 2021;7(12). 10.1016/j.heliyon.2021.e08526 PMC8661021 34934842

[22] Chilambe K, Mulubwa C, Zulu JM, Chavula MP. Experiences of teachers and community-based health workers in addressing adolescents’ sexual reproductive health and rights problems in rural health systems: a case of the RISE project in Zambia. BMC Public Health. 2023;23(1):335. 36793027 10.1186/s12889-023-15199-5 PMC9930354

[23] Chavula MP, Zulu JM, Hurtig AK. Factors influencing the integration of comprehensive sexuality education into educational systems in low- and middle-income countries: a systematic review. Reprod Health. 2022;19(1):196. 36175901 10.1186/s12978-022-01504-9 PMC9524136

