# Supplementary material for: Exploring the barriers, facilitators, and opportunities to enhance uptake of sexual and reproductive health, HIV and GBV services among adolescent girls and young women in Zambia: a qualitative study
Source: BMC Public Health. 2024 Aug 13;24:2191. doi: 10.1186/s12889-024-19663-8 (PMC11321158; doi:10.1186/s12889-024-19663-8)
Supplement: Supplementary file 1 — Supplementary Material 1 [file 12889_2024_19663_MOESM1_ESM.docx]

**FORMATIVE ASSESSMENT OF HIV, GBV AND SEXUAL AND REPRODUCTIVE HEALTH STATUS AMONG ADOLESCENT GIRLS AND YOUNG WOMEN IN ZAMBIA**

**Key Informant Interview Guide:**

**National Policy Makers of HIV, GBV AND SRH Services**

**FOR OFFICIAL USE ONLY**

Study Location: **Lusaka**

Date of interview: ______/ _______/ ____________

Venue: _______________________________

Designation of Respondent: ____________________

Interviewer _______________________

Time started: ________________ Time ended: _________________

**Key Informant Category**

Policy maker:

Key informant interview identifier: _______________________________

**Note: The key informant identifier** should be composed of the initials “KII” followed by the respondent’s designation (maximum 3 digits), date of interview in the format ***dd/mm/yy*** and informant’s number (3 digits) assigned cumulatively. For example, if the first respondent is a Coordinator for Reproductive Health, interviewed on October 15^th^, 2022; this informant’s identifier should be in the form: **KII/CRH//15/10/2021.**

***Interviewer:* Please obtain any additional details about the informant, e.g. the informant’s name and telephone contacts. These should be kept separately for any follow-up interviews that may be deemed necessary after the initial contact (Allow participants fill in an attendance list indicating their name and telephone contacts).**

**Introduction- Department/ Organization**

a) Kindly tell me about your work in relation with your role in delivery of HIV, GBV AND SRH Services among adolescent girls and young women?

**Section A: Service Availability**

**(Interviewer note:** *Please for all questions seeking HIV, GBV, and SRH related information, the responses for each should be captured separately)*

1. Please tell me about the provision of HIV, GBV, and other SRH services to AGYW in Zambia. What

- HIV services are provided and what mechanisms/approaches are used to deliver these services to AGYW?
- SRH services are provided and what mechanisms/approaches are used to deliver these services to AGYW?
- GBV services are provided and what mechanisms/approaches are used to deliver these services to AGYW?

1. What are the standardized HIV, GBV, and other SRH implementation policies and guidelines for health workers and implementers to use in delivery of these services?

- HIV policy guidelines
- SRH Policy Guidelines
- GBV Policy Guidelines

1. How are these guidelines and policies disseminated to the health facilities and other service delivery points?
2. How are these guidelines and policies made available?
   - At health facilities and other service delivery points?
   - Among AGYW implementers?
3. How do you ensure that the health facility and key implementing partner staff strictly follow the guidelines in providing HIV, GBV and SRH services to AGYW? Please explain your response.
4. What is your view of the capacity of health workers and implementers to deliver an integrated package of HIV, GBV, and other SRH services? *Probe on technical (training) and infrastructural capacity among others.*
5. *Do the current HIV, GBV and SRH services and health systems address issues specific to AYPLHIV? Explain.*
6. What role do you think Adolescent and Young People Living with HIV (AYPLHIV) can play in the provision of HIV, GBV and SRH services?

- How can they be meaningfully involved?
- What about the family support groups that exist at some health facilities?

1. What are the gaps in delivery of HIV, GBV, and SRH services to AYPLHIV? How can they be addressed?

**Section B: Uptake and Utilization of HIV, GBV, and SRH Services**

1. In your opinion, what challenges do AGYWs face in accessing HIV, GBV and SRH services?

- How do these challenges differ by age of the AGYW – i.e. 10-14, 15-19, or 20-24 years?
- *Probe for stigma in accessing SRH; systems, logistics challenges etc.*
- How can these challenges be overcome?

1. What **age-appropriate** HIV, GBV, and SRH services do you think should be provided to adolescent girls and young women? How should EACH of these services be provided?

- HIV services
- GBV services and support structures (one stop centers, police
- SRH at community and health facility level

1. What HIV, GBV, and SRH service options would you not recommend for AGYW in general and AYPLHIV in particular? Please explain:

- System requirements needed to meet HIV, GBV, and SRH needs for adolescent girls and young women living with HIV at service delivery points? *Probe for personnel (numbers and training, supplies, information management systems etc.)*

1. What are the limitations/barriers in addressing the challenges that adolescent girls and young women living with HIV face in accessing HIV, GBV, and SRH services?

- *Probe for issues of stigma in accessing STI services by HIV positive adolescent girls and young women,*
- *Probe infrastructure, human resources, logistics, disclosure, system challenges,*
- *Probe loss to follow-up, school dropout and providers’ attitudes, etc.*

**Section C: HIV, GBV and SRH Service Integration**

Now, let us talk about the integration of SRH services in HIV/STI, and GBV.

1. In your view, how far has the country gone towards integrating:

- HIV/STI with adolescent SRH services? What are the successes? What do you think has not been done? In your opinion, which services should be integrated together? Where should this integration happen?
- GBV with adolescent SRH services? What are the successes? What do you think has not been done? In your opinion, which services should be integrated together? Where should this integration happen?

1. What in particular SRH-HIV-STI services may be more difficult to integrate? Please explain:

- Probe for counseling for family planning,
- Probe for STI diagnosis and treatment, Sexual health/hygiene
- Probe for Ate Natal Care (ANC), postnatal care, delivery, prevention of mother to child transmission (PMTCT)
- Probe for cervical cancer screening,
- Probe for consultations on sexuality and fertility or infertility issues)

1. What mechanism do you have for the collection of HIV, GBV and SRH data and submission to the national level?

- What comments do you have on the existing mechanisms?
- What suggestions do you have to improve the current systems?

1. Finally, integration of services has been touted (advertised) as the best model to improve service delivery in most settings since the client can receive multiple services within the same facility. However, full-scale is yet to be achieved in Zambia.
   - What activities on SRH, GBV and HIV you are integrating? What suggestions do you have overall towards improving integration of HIV, GBV, and SRH services for AGYW in general and AYPLHIV in particular?
   - What models of HIV/STI, GBV and SRH integration would you recommend? Please provide reasons for your response.

**Section D: Collaboration IN HIV, GBV and SRH Service.**

Now, let us focus on collaboration in HIV, GBV and SRH service delivery.

1. Which actors / stakeholders are involved in implementing of HIV, GBV and SRH service:

- HIV, Services in this province/ district? (probe for NGOs, youth led organisations, churches etc.)
- GBV Services in this province/ district? (probe for NGOs, churches etc.)
- SRH Services in this province/ district? (probe for NGOs, churches etc.)

1. What roles do these actors/ stakeholders play?
2. What is the benefit / value of having these stakeholders?
3. How is the coordination process of HIV, GBV and SRH Services across partners / stakeholders done?
   - HIV, Services in this province/ district?
   - GBV Services in this province/ district?
   - SRH Services in this province/ district?
4. What has worked well with this stakeholder coordination/ partnership?
5. What are the gaps in the partnership/ coordination?
6. Please tell me if there are any stakeholders that have been left? If so, why? What be done to bring them on board?
7. What can be done to improve the collaboration of stakeholders in implementing HIV, GBV and SRH Services?
   - HIV, Services in this province/ district?
   - GBV Services in this province/ district?
   - SRH Services in this province/ district?
