# Supplementary material for: Exploring the barriers, facilitators, and opportunities to enhance uptake of sexual and reproductive health, HIV and GBV services among adolescent girls and young women in Zambia: a qualitative study
Source: BMC Public Health. 2024 Aug 13;24:2191. doi: 10.1186/s12889-024-19663-8 (PMC11321158; doi:10.1186/s12889-024-19663-8)
Supplement: Supplementary file 2 — Supplementary Material 2 [file 12889_2024_19663_MOESM2_ESM.docx]

**FORMATIVE ASSESSMENT OF HIV, GBV AND SEXUAL AND REPRODUCTIVE HEALTH STATUS AMONG ADOLESCENT GIRLS AND YOUNG WOMEN IN ZAMBIA**

**Key Informant Interview Guide:**

**District Implementers of HIV, GBV AND SRH Services**

**FOR OFFICIAL USE ONLY**

Study Location: **Mazabuka; Chongwe; Mongu**

Date of interview: ______/ _______/ ____________

Venue: _______________________________

Designation of Respondent: ____________________

Interviewer _______________________

Time started: _______________________ Time ended: _________________

**Key Informant Category:** HIV, GBV AND SRH Services Implementer

Key informant interview identifier: _______________________________

**Note: The key informant identifier** should be composed of the initials “KII” followed by the respondent’s designation (maximum 3 digits), date of interview in the format ***dd/mm/yy*** and informant’s number (3 digits) assigned cumulatively. For example, if the first respondent is a Principal Vocational Education College, interviewed on October, 18^th^ 2022; this informant’s identifier should be in the form: **KII/P/VE/01/18/10/2021.**

***Interviewer:* Please obtain any additional details about the informant, e.g., the informant’s name and telephone contacts. These should be kept separately for any follow-up interviews that may be deemed necessary after the initial contact.**

**DISTRICT IMPLEMENTERS –** District Community Development Officer, Guidance and Counselling Teacher (School), Adolescent Focal Point (Health Facility), Adolescent Focal Point (District), District HIV coordinator AND Partners [NGOs i.e., CIDRZ, Future Life Now, World Vision Zambia, GIZ etc.]

**Introduction- Department/ Organization**

a) Kindly tell me about your work in relation with your role in delivery of HIV, GBV AND SRH Services among adolescent girls and young women?

**Section A: Service Availability**

**(Interviewer note:** *Please for all questions seeking HIV, GBV, and SRH related information, the responses for each should be captured separately)*

1. What are the standardized HIV, GBV, and other SRH implementation policies and guidelines for health workers and implementers to use in delivery of these services?

- HIV policy guidelines
- SRH Policy Guidelines
- GBV Policy Guidelines

1. How are these guidelines and policies disseminated to the health facilities and other service delivery points?
2. How these guidelines and policies are made available?
   - At health facilities and other service delivery points?
   - Among AGYW implementers?
3. Please tell me about the provision of HIV, GBV, and other SRH services to AGYW in Zambia. What

- HIV services are provided and what mechanisms/approaches are used to deliver these services to AGYW?
- SRH services are provided and what mechanisms/approaches are used to deliver these services to AGYW?
- GBV services are provided and what mechanisms/approaches are used to deliver these services to AGYW?

1. How do you ensure that the health facility and key implementing partner staff strictly follow the guidelines in providing HIV, GBV and SRH services to AGYW? Please explain your response.
2. What is your view of the capacity of health workers and implementers to deliver an integrated package of HIV, GBV, and other SRH services? *Probe on technical (training) and infrastructural capacity among others.*
3. *Do the current HIV, GBV and SRH services and health systems address issues specific to AYPLHIV? Explain.*
4. What role do you think Adolescent and Young People Living with HIV (AYPLHIV) can play in the provision of HIV, GBV and SRH services?

- How can they be meaningfully involved?
- What about the family support groups that exist at some health facilities?

1. What are the gaps in delivery of HIV, GBV and SRH services to AYPLHIV? How can they be addressed?
2. What HIV, GBV and SRH related services do you offer to AGYW in general and AYPLHIV in particular within this district? (*Probe: Probe for linkages for referral if the services are not provided onsite)*
   - HIV, Services in this province/ district?
   - GBV Services in this province/ district?
   - SRH Services in this province/ district?
3. In your opinion, can all AGYW (regardless of age) access the HIV, GBV and other SRH services that they need? If yes, why? If not, why not? (*probe for access to* ***age-appropriate (age of consent)*** *HIV, GBV and SRH services for AGYW)*
   - HIV, Services in this province/ district?
   - GBV Services in this province/ district?
   - SRH Services in this province/ district?
4. Are there any HIV, GBV and other SRH-related services that AGYW would wish to receive but which you do not provide in this district? If yes, what are these services and why don’t you provide them?
   - HIV, Services in this province/ district?
   - GBV Services in this province/ district?
   - SRH Services in this province/ district?
5. What gaps exist in delivery of HIV, GBV and SRH services to AGYW in this district? What, in your opinion, should be done in order to increase access to and utilization of HIV, GBV and other SRH services among AGYW – both in- and out-of-school? (*Probe for methods appropriate for different age categories of 10-14, 15-19 and 20-24 years*)
   - HIV gaps in services in this province/ district?
   - GBV gaps in services in this province/ district?
   - SRH gaps in services in this province/ district?

**Section B: Accessibility and Utilization of HIV, GBV and SRH services**

1. How is the provision of HIV, GBV and SRH services to AGYW organized (structured, programmed) in this district? How favorable is this approach in helping AGYW to access and use these services? Please explain.
2. In your opinion, what challenges do AGYW in your community face in accessing HIV, GBV and SRH services? *(Probe for stigma in accessing SRH; systems, logistics challenges etc.).*
   - How do these challenges differ by age of the AGYW – i.e. 10-14, 15-19, or 20-24years?
   - *Probe for stigma in accessing SRH;* HIV, GBV, *health systems, trust, logistics challenges etc.*
   - How can these challenges be overcome?

1. What **age-appropriate** HIV, GBV, and SRH services would you recommend for AGYW in this district? *Probe for how the AGYWs’ family or community could be meaningfully involved to improve access and utilization of HIV, GBV, and SRH services.*
   - HIV services
   - GBV services and support structures
   - SRH at community and health facility level
2. Are there any HIV, GBV, and SRH services you would not recommend for AGYW? Please explain.
   - HIV services
   - GBV services
   - SRH at community and health facility level
3. In this district, what limitations/barriers do you face while providing HIV, GBV, and SRH services to AGYW? (*Probe for issues of stigma in accessing services by HIV positive adolescent girls and young women, the timing (for AGYW in-school), infrastructure, human resource, logistics, disclosure, providers’ attitudes, etc.)*
4. What are the limitations/barriers in addressing the challenges that adolescent girls and young women living with HIV face in accessing HIV, GBV, and SRH services?

- *Probe for issues of stigma in accessing STI services by HIV positive adolescent girls and young women,*
- *Probe infrastructure, human resource, logistics, disclosure, system challenges,*
- *Probe loss to follow-up, school dropout and providers’ attitudes, etc.*

1. What mechanism do you have for collection of HIV, GBV and SRH data for AGYWs and submission to the national level? What are your comments on this mechanism? How do you think it can be improved?

**Section C: HIV, GBV and SRH Service Integration**

*Now, let us focus on integration of HIV, GBV in SRH service delivery.*

1. In your view, how far has your district gone towards integrating HIV/STI and GBV with adolescent sexual and reproductive health services?

- Probe: What are the successes?
- Probe: What do you think has not been done?
- Probe: In your opinion, which services should be integrated together?
- Probe: Where should this integration happen?

1. Do you currently provide integrated HIV/STI, GBV and SRH services? If so;

- Probe: What models of service integration do you use?
- Probe: What experiences have you gained in the process of integrating these services?
- Probe: What challenges have you experienced?
- Probe: How would you advise other programs with regard to integration of HIV/STI, GBV and SRH services?

1. What in particular SRH-HIV-STI, GBV and SRH services may be more difficult to integrate? Please explain:

- Probe for counseling for family planning,
- Probe for STI diagnosis and treatment, Sexual health/hygiene
- Probe for ANC, postnatal care, delivery, PMTCT,
- Probe for cervical cancer screening,
- Probe for consultations on sexuality and fertility or infertility issues)

1. Finally, in some health facilities, integration of services has already happened while in others, it is yet to happen. What suggestions do you have overall towards improving integration?

- Probe: HIV services for AGYW in general and AYPLHIV in particular
- Probe: GBV services for AGYW in general and AYPLHIV in particular
- Probe: SRH services for AGYW in general and AYPLHIV in particular

**Section D: Collaboration in delivering HIV, GBV and SRH Service.**

*Now, let us focus on collaboration in HIV, GBV and SRH service delivery among AGYW*

1. Which actors / stakeholders in the district are involved in implementing:

- HIV services (probe for NGOs, churches, peer educators, young led organization etc.)
- GBV services (probe for NGOs, churches peer educators, young led organization etc.)
- SRH services (probe for NGOs, churches etc.)

1. What roles do these actors/ stakeholders play *HIV, GBV and SRH* services AGYW?

1. What is the benefit / value of having these stakeholders?
2. How is the coordination process of HIV, GBV and SRH Services across partners / stakeholders done?
   - HIV, Services in this province/ district?
   - GBV Services in this province/ district?
   - SRH Services in this province/ district?
3. What has worked well with this stakeholder coordination/ partnership?
4. What are gaps in the partnership/ coordination?
5. Please tell me if there are any stakeholders that have been left? If so, why? What can have done to bring them on board?
6. What can be done to improve the collaboration of stakeholders in implementing HIV, GBV and SRH Services for AGYW in general and adolescent girls and young people living with HIV?
   - HIV, Services in this province/ district?
   - GBV Services in this province/ district?
   - SRH Services in this province/ district?

Is there anything that you want to add on all what you have said before we end our interview?

Thank you for your participation

**END OF INTERVIEW**
