# Supplementary material for: Exploring the barriers, facilitators, and opportunities to enhance uptake of sexual and reproductive health, HIV and GBV services among adolescent girls and young women in Zambia: a qualitative study
Source: BMC Public Health. 2024 Aug 13;24:2191. doi: 10.1186/s12889-024-19663-8 (PMC11321158; doi:10.1186/s12889-024-19663-8)
Supplement: Supplementary file 3 — Supplementary Material 3 [file 12889_2024_19663_MOESM3_ESM.docx]

**FORMATIVE ASSESSMENT OF HIV, GBV AND SEXUAL AND REPRODUCTIVE HEALTH STATUS AMONG ADOLESCENT GIRLS AND YOUNG WOMEN IN ZAMBIA**

**In-Depth Interview Guide**

**AGYW living with HIV, and/ or for AGYW involved in commercial sex worker, GBV survivors, the married, those who gave birth by age 15 and those currently pregnant.**

**FOR OFFICIAL USE ONLY**

Study Location: **Mazabuka; Chongwe; Mongu**

Date of interview: ______/ _______/ ____________

Name of district: _______________________________

Venue: ____________________

Language of interview: _______________________

Time interview started: ________________ Time interview ended: _________________

In-depth interview identifier: _____________________________________

**Note: The in-depth interview participant’s identifier** should be created as follows: Type the initials - “IDI” – followed by the name of the Village/Town/health facility where the interview has taken place (first 3 digits), followed by the date of interview in the format ***dd/mm/yy*** and the participant’s number (3 digits) assigned cumulatively. The participant’s identifier should end with a code showing the type of client as explained below. For example, if the first respondent is interviewed on October 18, 2021 in Kabwe district; this participant’s identifier should be in the form: **I**DI/KD/18/10/20/2O21/CSW, where CSW= Commercial Sex Worker. Please use: LWH = Living with HIV; PSD = Pregnant School Dropout, SGBV= Survivor of GBV, CM= currently married, GB15= Gave birth by age 15, LBP= Local Businessperson, etc. at the end of each participant’s number to designate the type of participant interviewed.

***Interviewer:* Please obtain any additional details about the participant, e.g. his/her name and telephone contacts should be kept separately for any follow-up interviews that may be deemed necessary after the initial contact.**

**PARTICIPANTS SOCIO-DEMOGRAPHIC INFORMATION**

1. Age-group

10-14 1

15-19 2

20-24 3

1. Gender

Male 1

Female 2

1. School status

In-school 1

Out of school 2

1. Marital status

Single/never married 1

Currently married 2

Divorced 3

Widowed 4

1. Education level

No education 1

G1-G4 2

G5-G7 3

G1-G4 4

G5-G6 5

University 6

Other (specify)______________ 7

1. Religious affiliation

Catholic 1

Protestant 2

Muslim 3

Pentecostal 4

SDA 5

Other (specify) ______________ 6

1. Occupation

Not working 1

Small scale business 3

Formal employment 4

1. HIV & ART Status

HIV positive on ART 1

HIV positive not on ART ` 2

HIV Negative 3

1. GBV Status

GBV survivor 1

Not a GBV survivor 2

**Guidance to the interviewer:** This guide has two parts; **Part 1** is to be used with adolescent girls and young women living with HIV and **Part 2** is for those out of school, those doing commercial sex work, gender-based violence survivors, the Married, those who gave birth by age 15 and those currently pregnant.

**PART 1 (ONLY AGYW LIVING WITH HIV)**

**(Interviewers note:** *Please for all questions seeking HIV, GBV, and SRH related information, the responses for each should be captured separately)*

**Section A: General Questions**

1. Let’s us begin by talking about what you understand by the terms; HIV, GBV and SRH care (*Probe for the different services they you know)*
2. As someone who is living with HIV, what challenges do you undergo while accessing HIV, GBV or SRH services?
3. Where do you access HIV, GBV and SRH services they need? (probe for the costs involved and preferred time for respondent or someone of the same category (i.e. AGYW living with HIV) to go for the services)
4. Tell me about your experiences in HIV, GBV and SRH services. What have you liked or liked? What challenges have you faced? Probe for comforting, management or rehabilitation?

**Section B: Service availability, uptake and utilization**

1. What process did you go through for getting into the HIV care?
2. What HIV, GBV and SRH services are available in your community? (*Probe for linkages for referral if the services are not provided onsite)*
3. What are your experiences in accessing HIV care?
4. Are there any HIV, GBV or SRH services in general that you would prefer to use but are not easy to find in your community, nearby health facility or where you are referred? (*probe which ones*)
5. How would you like HIV, GBV and SRH services to be organized for AGYW like you (interviewer to state the appropriate category) to easily access and utilize them in your community?
6. About the last time you visited the health facility; did you receive the HIV, GBV or SRH services you needed? (*If respondent receive HIV, GBV and SRH services, ask: what services they receive, were they satisfied with the services?*
7. Of the HIV, GBV or SRH services you accessed,
   1. What services did you like most and why?
   2. What services didn’t you like and why?
   3. How you what the services to be provided?
8. How do you get information or services about HIV, GBV and SRH services? (*Probe if there exist new technologies such as mobile apps where young people receive messages about HIV and SRH services)*
9. ***Section C: Recommendations***
10. *Do you have any suggestions on how to improve provision of HIV, GBV and SRH care for adolescents and young people living with HIV in your area?*
11. *Any questions/comments you would like to make regarding this study?*
12. ***Section D: Decision making process and factors for choice of dropping out of school***
13. *Let’s talk about schooling experience.*
14. *Tell me how you started school? Probe for type of school (Public or Private: level of education, distance from home to school)*
15. *How did you come to stop schooling?*
16. *Why did you stop going to school? (Probe on factors such as factors that led to dropping out of school)*
17. *Share with me the kind of work that you have been involved in after dropping out of school?*
18. *Other than the reasons you have mentioned above, what else could have influenced you to choose to do what you are currently doing? (for only those involved in sex work and those currently married, Probe for peer influence, socio-economic factors, and information bias, availability etc)*
19. *If you were to be supported to re-engage in school, at your previous school (in what grade) to do another preferred job, would you be willing to go back to school? If No, Why? If yes, (probe which course/program or job opportunity most preferred)*
20. *How did you feel after choosing to SW business?*
21. *Do you have any regrets for choosing SW business? Please explain more*

**PART 2 A: For AGYW involved are sex worker, gender-based violence survivors, the Married, those who gave birth by age 15 and those who are currently pregnant.**

**(Interviewer note:** *Please for all questions seeking HIV, GBV, and SRH related information, the responses for each should be captured separately)*

**Section A: General Questions**

1. (**Interviewer to take care while selecting respondent’s category to avoid misclassification):** As someone who is engaged in sex work**/**As someone who is currently married**/**As someone who is currently pregnant/Gave birth by age 15/As a survivor of gender based violence, what HIV, GBV or SRH challenges have you ever experienced?
2. Let’s begin by talking about what you understand by the terms ‘HIV, GBV and SRH care (Probe *for the different services they you know)*
3. If you are faced with an HIV, GBV and SRH challenge (for example if you are raped/sexually assaulted, beaten, forced into marriage etc.), where do you go for comforting, management or rehabilitation? *(probe for type and ownership of facilities and the services offered)*
4. Where in this area can AGYW out-of school get HIV, GBV and SRH services they need? *(Probe for the costs involved in getting the services, distance and preferred facility opening hours to go for the services)*

**Section B: integration in accessing HIV, GBV and SRH services**

1. In your experience, what HIV, GBV and SRH services do you always receive at your preferred health facility?
2. What is the process of getting into care for HIV, GBV and SRH?
3. What are your good and bad experiences about HIV, GBV and SRH services that you accessed?
4. Are there any HIV, GBV or SRH services in general that you would like to use but are not provided by facilities in your community or where you are referred? *(probe: which services are missing, yet preferred)*
5. How would you like HIV, GBV and SRH services to be organized for *AGYW like you (interviewer to state the appropriate category for example,* *AGYW involved in sex worke, GBV survivors, the Married, those who gave birth by age 15 and those currently pregnant)* to easily access and utilize them in your community?
6. Do you have any suggestions on how to improve the provision of HIV, GBV and SRH service access and utilization for AGYW like you in your area?
7. Any questions/comments you would like to make regarding this interview?

**Section C: Decision making process and factors for choice of dropping out of school**

Let’s talk about schooling experience;

1. Tell me how you started school? Probe for type of school *(Public or Private: level of education, distance from home to school)*
2. How did you come to stop schooling?
3. Why did you stop going to school? (*Probe on factors such as factors that led to dropping out of school)*
4. Share with me the kind of work that you have been involved in after dropping out of school?
5. Other than the reasons you have mentioned above, what else could have influenced you to choose to do what you are currently doing? *(****for only those involved in sex work and those currently married, Probe for peer influence, socio-economic factors, and information bias, availability etc****)*
6. If you were to be supported to re-engage in school, at your previous school (in what grade) to do another preferred job, would you be willing to go back to school? If No, Why? If yes, (*probe which course/program or job opportunity most preferred*)
7. How did you feel after choosing to SW business? How do you feel about the work you do? Probe for benefits and challenges.

**Section D: Recommendations**

1. Do you have any suggestions on how to improve provision of HIV, GBV and SRH care for AGYW in your area?
2. Do you have any suggestions on how second chance education could be structured for adolescents and young people who dropped out of school?
3. Any questions/comments you would like to make regarding this interview?
